# The TORC1 inhibitor Nprl2 protects age-related digestive function in *Drosophila*

**DOI:** 10.18632/aging.102428

**Published:** 2019-11-11

**Authors:** Junmeng Xi, Jiadong Cai, Yang Cheng, Yuanyuan Fu, Wanhong Wei, Zhenbo Zhang, Ziheng Zhuang, Yue Hao, Mary A. Lilly, Youheng Wei

**Affiliations:** 1 College of Bioscience and Biotechnology, Yangzhou University, Yangzhou, China; 2 Joint International Research Laboratory of Agriculture and Agri-Product Safety, The Ministry of Education of China, Yangzhou University, Yangzhou, China; 3 Institute of Reproduction and Metabolism, Yangzhou University, Yangzhou, China; 4 Reproductive Medicine Center, Department of Obstetrics and Gynecology, Shanghai General Hospital, Shanghai Jiao Tong University School of Medicine, Shanghai, China; 5 School of Pharmaceutical Engineering and Life Sciences, Changzhou University, Changzhou, China; 6 Key Laboratory of Pollinating Insect Biology, Ministry of Agriculture, Beijing, China; 7 Cell Biology and Neurobiology Branch, National Institute of Child Health and Human Development, National Institutes of Health, Bethesda, MD 20892, USA

**Keywords:** Target of rapamycin complex 1 (TORC1), Nprl2, Drosophila, crop, gut

## Abstract

Aging and age-related diseases occur in almost all organisms. Recently, it was discovered that the inhibition of target of rapamycin complex 1 (TORC1), a conserved complex that mediates nutrient status and cell metabolism, can extend an individual’s lifespan and inhibit age-related diseases in many model organisms. However, the mechanism whereby TORC1 affects aging remains elusive. Here, we use a loss-of-function mutation in *nprl2*, a component of GATOR1 that mediates amino acid levels and inhibits TORC1 activity, to investigate the effect of increased TORC1 activity on the occurrence of age-related digestive dysfunction in *Drosophila*. We found that the *nprl2* mutation decreased *Drosophila* lifespan. Furthermore, the *nprl2* mutant had a distended crop, with food accumulation at an early age. Interestingly, the inappropriate food distribution and digestion along with decreased crop contraction in *nprl2* mutant can be rescued by decreasing TORC1 activity. In addition, *nprl2*-mutant flies exhibited age-related phenotypes in the midgut, including short gut length, a high rate of intestinal stem cell proliferation, and metabolic dysfunction, which could be rescued by inhibiting TORC1 activity. Our findings showed that the gastrointestinal tract aging process is accelerated in *nprl2*-mutant flies, owing to high TORC1 activity, which suggested that TORC1 promotes digestive tract senescence.

## INTRODUCTION

Aging involves the progressive loss of physiological functions in all organs and tissues and therefore, increases vulnerability to death [1]. Age-related diseases, such as cancer, osteoporosis, and diabetes, are associated with an increase in cellular senescence [2, 3]. In fact, many age-related diseases occur in young individuals in response to genetic and environmental insults [3]. Thus, defining the genes and pathways that control aging is essential to improve the treatment of age-related diseases.

Target of rapamycin (TOR) signaling is strongly implicated in the aging processes of diverse organisms, including yeast, worms, flies, and mammals [4]. TOR is an evolutionally conserved serine/threonine kinase that forms the catalytic subunit in two multi-protein complexes, TORC1 and TORC2, which have distinct structures and functions in metabolism [5]. TORC1 is sensitive to rapamycin and plays a central role in cell growth. It controls the balance between anabolism and catabolism in response to multiple inputs, including amino-acid availability, growth factor levels, and intracellular energy status. When nutrients are sufficient, TORC1 is recruited to lysosomal membranes by the Rags GTPase, where it comes in contact with the Rheb GTPases, which promote TORC1 kinase activity [6, 7]. Activated TORC1 phosphorylates multiple downstream effectors, such as S6K and 4E-BP, to increase the synthesis of proteins, lipids, and nucleotides. In contrast, when nutrients are scarce, TORC1 is inactive and this triggers catabolic metabolism and autophagy to provide energy for cell survival. Thus, cells can rapidly fine-tune their metabolic state in response to the environment by modulating the activity of TORC1 [8].

In *Drosophila*, Rag GTPase is comprised of a heterodimer of RagA and RagC [6, 7]. When nutrients are sufficient, RagA binds GTP and RagC binds GDP to promote the recruitment of TORC1 to lysosomes, where the GTP-loaded form of Rheb GTPase binds and directly stimulates TORC1 kinase activity [9]. Two complexes, known as TSC and GATOR1, function as GAPs (GTPase-activating proteins) towards Rheb and RagA, respectively and thus, act as inhibitors of TORC1 activity [10, 11]. TSC comprises TSC1 and TSC2 and is repressed by growth factors and high cellular energy levels [12]. The GATOR1 complex, composed of Nprl2, Nprl3, and DEPDC5/Iml1, is inactivated by amino acids [13, 14]. In *Drosophila* and mammals, depleting any of the GATOR1 or TSC complex components results in a dramatic increase in TORC1 activity and cell size [13, 14]. Thus, TSC and GATOR1 cooperatively control TORC1 activity. Genetic manipulation or pharmacological treatment that reduces TORC1 activity promotes longevity in multiple model organisms, including yeast, *Drosophila*, and mice [15–17]. Although the relationship between decreased TORC1 activity and longevity is widely established, the mechanism remains unclear. Several models have been suggested as to how reduced TORC1 activity affects aging, including a reduction in global mRNA translation, increased autophagy, and the stimulation of stress-resistance pathways [3, 4].

One system that is affected by aging in humans is the digestive system or the gastrointestinal (GI) tract. The GI tract functions as a digestive system to absorb energy and nutrients from the environment and as a barrier epithelium to protect the animal from the exterior environment. Disruptions in this epithelial barrier negatively affect animal health and significantly decrease lifespan [18]. The human GI tract is divided into four distinct sections: the esophagus, stomach, small intestine, and large intestine (colon) [19]. They are separated from each other and control the movement of food or food residues from one section to another. During this process, the ingested food is digested, and the nutrients are absorbed. Finally, the remaining waste is expelled from the body as feces. Gastrointestinal motility is required to efficiently shuttle liquified food from the stomach into the intestine and thus, is essential to health [19, 20]. There are multiple changes associated with GI tract aging, including gastric motility, intestinal hyperplasia, and hormone secretion [21, 22]. Some clinical reports have shown increased morbidity associated with dysfunctional GI tract motility and gastric emptying in the aged population [20]. However, the underlying cellular and molecular mechanisms involved in the effect of aging on GI tract motility and gastric emptying are largely unknown.

The *Drosophila* digestive tract is divided into three discrete domains: foregut, midgut, and hindgut [23, 24]. The foregut, comprising the crop, esophagus, and cardia, serves as a storage site for ingested food [25, 26]. Functionally similar to the stomach, the crop is a structure consisting of a complex array of valves and sphincters that move food contents into the midgut. The midgut is a long tubular structure that is equivalent to the mammalian small intestine and is the main site for food digestion and nutrient absorption. The epithelial layer of the midgut mainly consists of two differentiated cell types - polyploid absorptive enterocytes (ECs) and hormone-secreting enteroendocrine (EE) cells, which originate from intestinal stem cells (ISCs) residing basally in the intestinal epithelium [27, 28]. Recent studies have established the *Drosophila* intestine as a model system to study intestinal tissue homeostasis, immune responses, and stress signaling [23, 24]. Damage to the intestinal epithelium can trigger ISC division and differentiation to replace lost cells and maintain tissue homeostasis [29]. The aging intestines exhibit increased proliferation of ISCs and reduced regeneration of epithelial cells, which leads to gut hyperplasia; metabolic disorders; barrier leakage; immune senescence; and ultimately, the death of the fly [30]. However, the mechanism of digestive dysfunction in aging flies remains elusive.

Here, we used mutations in the GATOR1 complex component, Nprl2, to explore how TORC1 activity promotes age-related physiological defects in the GI tract. Importantly, we found that crop emptying and GI tract motility defects in *nprl2-*mutant flies are phenocopied in aged wild-type flies. Moreover, we observed that the *nprl2*-mutant midgut displays age-related defects in gut morphology, ISC populations, metabolism, and immune responses. Our findings highlight the importance of TORC1 activity in aging and age-related GI tract disease and suggest that the *Drosophila* crop can be a useful model system to investigate the mechanisms of gastric emptying dysfunction in elderly individuals.

## RESULTS

### The *nprl2*-mutant flies display shorter lifespan

**Figure 1:**
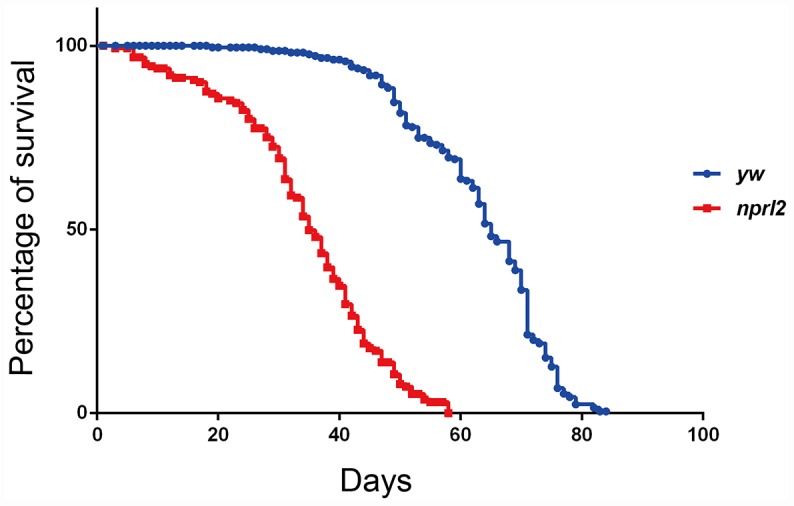
**The *nprl2* mutant had a decreased lifespan.** Newly hatched male flies were cultured on standard food and counted each day. Survival curves of *yw* (n = 205) and *nprl2^1^* (n = 154) flies are shown. Pairwise comparisons by the Mantel-Cox log rank test showed *P* < 0.0001.

Recently, the TOR pathway has emerged as an important modulator of aging [4]. Genetic and pharmacological inhibition of TORC1 activity extends lifespan and prevents multiple age-related diseases in several model organisms [17, 31–33]. Thus, we reasoned that mutations in *nprl2*, which have been previously shown to increase TORC1 activity, might affect *Drosophila* longevity [34]. To test this possibility, we compared the lifespan of *nprl2* null mutants, *nprl2^1^*, with the lifespan of the control stock, *yw,* under standard culture condition. As shown in Figure 1, the median lifespan of *nprl2^1^* flies (34 d) was markedly shorter than that of wild-type flies (64 d). Thus, mutations in *nprl2* resulted in significantly decreased *Drosophila* lifespan.

### Nprl2 is required for GI tract digestive function

**Figure 2:**
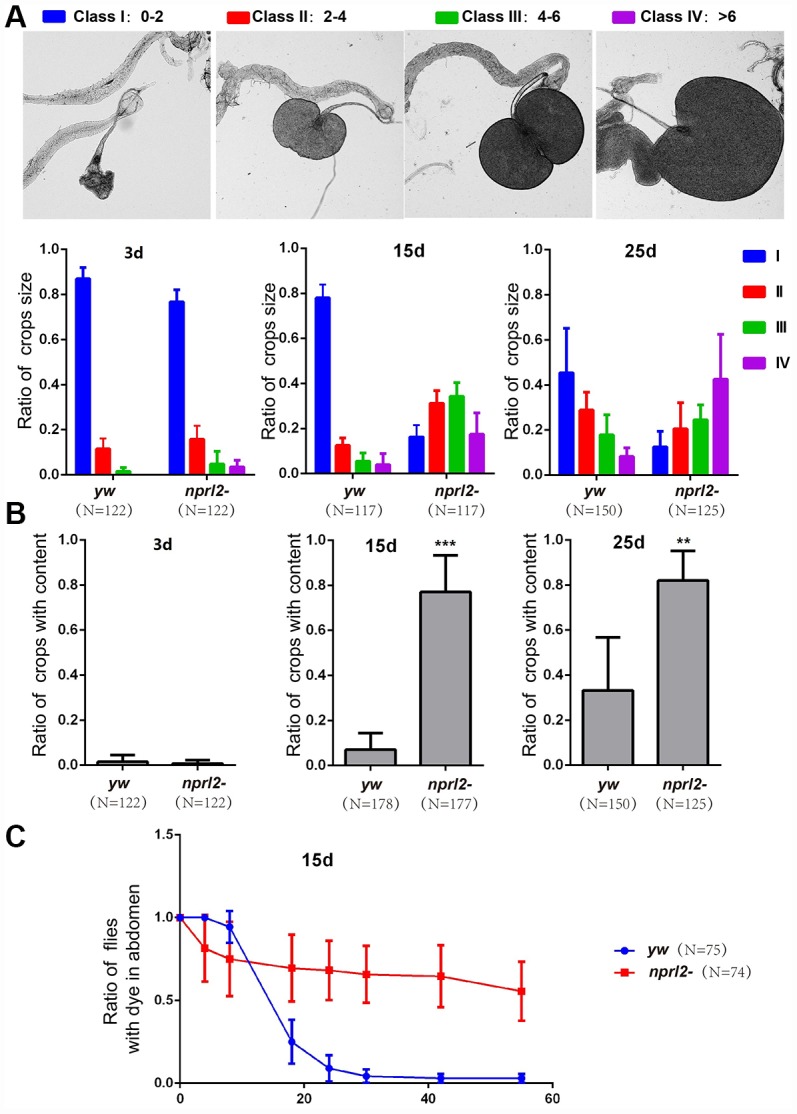
**Nprl2 plays important roles in *Drosophila* digestive function.** (**A** and **B**) Newly hatched *yw* and *nprl2^1^* flies were cultured on standard food for the indicated number of days and then dissected. (**A**) Crop sizes were grouped into four classes based on area (mm^2^). The ratio of different crop sizes was quantified. Error bars represent the SD from four independent experiments. (**B**) The ratio of flies with food content in their crops was quantified. Error bars represent the SD from four independent experiments. (**C**) 15 day-old flies were cultured on blue dyed food for 3 days and then transferred to standard food for the indicated time. The ratio of flies with blue dye in the digestive tube at each time point was calculated. Error bars represent the SD from three independent experiments. **P < 0.01, ***P < 0.001. N is the total number of flies used.

In humans, age contributes to decreased gastrointestinal tract motility and negatively affects GI tract digestive function, resulting in symptoms such as esophageal reflux, delayed gastric emptying, and constipation [19, 20]. Similarly, older monkeys have dramatically reduced intestinal motility [35]. We aimed to determine if *Drosophila* *nprl2* mutants, which appear to have accelerated aging, exhibit age-related gastric dysfunction. The *Drosophila* crop, a foregut organ that is functionally comparable to the mammalian stomach, is primarily a reservoir for food and nutrients [23, 36]. Wild-type flies rarely exhibit large or extended crops when they have free access to food [37]. In contrast, we found that *nplr2*-mutant flies often developed a swollen crop as they increased in age. To better define the extent of the distended crop phenotype, we scored the crops for size and placed them in one of four categories, with 1 being the smallest crop and 4 being the largest (Figure 2A). Young wild-type adults had a small crop size, with less than 2% of flies showing crop sizes in class 3 or 4. As adult flies aged, the propensity for a large crop size increased, with approximately 10% of wild-type adults exhibiting a large crop size (class 3 or 4) at 15 d. This number rose to approximately 20% at 25 d, suggesting that age promoted crop distension in wild-type flies (Figure 2A). Consistent with the hypothesis that mutations in *nprl2* accelerate the aging process, at 15 d, 50% of *nprl2*-mutant adults had a large crop size (class 3 or 4), which was nearly a five-fold increase relative to control adults. With the age increased, approximately 70% of *nprl2*-mutant adults displayed large crop at 25 d. As the crops of *nprl2* mutants were small at 3 d (Figure 2A), we reasoned that the large crop size seen at 15 d resulted from increased food storage and/or retention. To test this hypothesis, we examined the percentage of *nprl2*-mutant and wild-type adults that clearly contained food in their crops. At 3 d, both control and *nprl2*-mutant flies had minimal food content in their crops. Consistent with the above findings that *nprl2*-mutant flies had larger crops at 15 and 25 days, the percentage of *nprl2*-mutant flies with crop food retention was significantly higher than the number of control flies with food retention (Figure 2B). To determine whether the distended crop was resulted from cell size increased in *nprl2* mutant, we compared the crops that place in class I or class III in both control and *nprl2* mutant flies (Supplementary Figure 1). Interestingly, the cell sizes between *yw* and *nprl2* that place in the same class were similar. However, the cell sizes between small and large crops were obviously different, even the flies were same genotype. To confirm that the increased crop size and food accumulation in *nprl2*-mutant flies resulted from the loss of Nprl2, we overexpressed HA-tagged Nprl2, using the Ubi-GAL4 driver, in the *nprl2*-mutant background and found that the expression of Nprl2 fully rescued the crop size and food accumulation defects in 15-day-old *nprl2* mutants (Supplementary Figure 2).

Although the *Drosophila* esophagus is directly connected to the proventriculus of the midgut, the ingested food is first diverted to the crop and is subsequently pumped into the midgut, rather than entering the midgut directly [36]. Thus, the crop plays a critical role in regulating food passaging. The abnormal size and amount of food accumulation in the crops of *nprl2-*mutant flies may be related to slow food passaging. To test this hypothesis, we fed flies with food that contains a non-absorbable blue dye (FD&C blue dye no. 1) and tracked the time taken to expel the dye. As shown in Figure 2C, less than 10% of control flies had dye in their digestive tracts after 24 hours. However, more than 50% of the *nprl2*-mutant flies still contained blue dye in their digestive tracts after 60 hours. Thus, crop (gastric) emptying was much slower in *nprl2*-mutant flies (Figure 2C). Taken together, our data indicated that Nprl2 is critical for the maintenance of digestive function in *Drosophila*.

### The *nprl2*-mutant flies display GI tract motility defects

**Figure 3:**
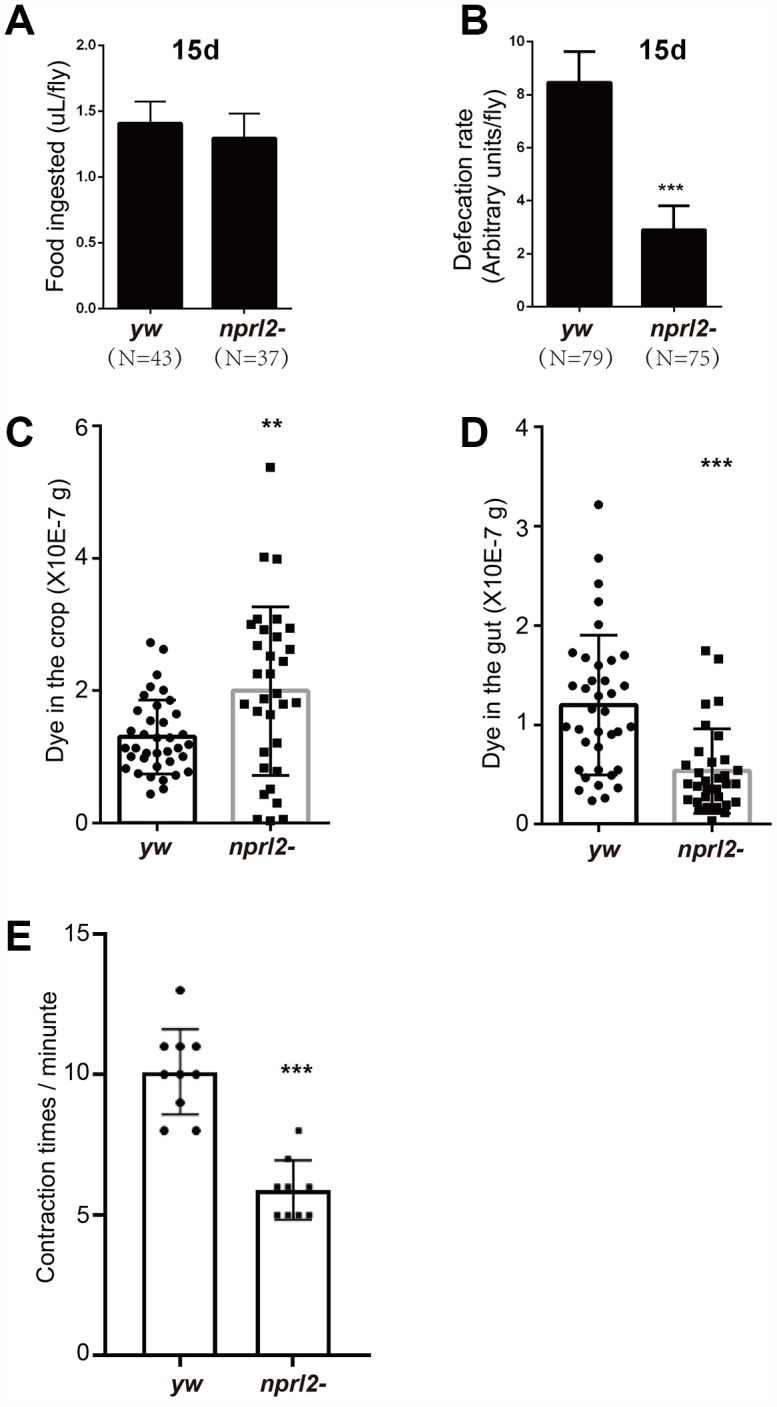
***nprl2*-mutant flies displayed GI tract motility defects.** Fifteen-day-old *yw* and *nprl2^1^* males were used for the following analyses. (**A**) CAFE assay to quantify food ingestion. Error bars represent the SD from eight independent experiments. (**B**) Defecation rate assay to quantify food expulsion. Error bars represent the SD from five independent experiments. (**C** and **D**) Flies were starved in complete starvation medium (PBS with 1% agarose) for 10 hours and then fed dyed food for 30 min. The dyed food concentration in crops and guts was determined using a spectrophotometer. (**E**) The contraction rate of the crop was measured in dissected flies. Error bars represent the SD of the indicated number of data points. **P < 0.01, ***P < 0.001. N is the total number of flies used.

The phenotypes of distended crop, food storage, and prolonged food passage through the digestive tract found in *nprl2*-mutant flies may be caused by either excessive food ingestion or slow food digestion. To determine which of these two processes was involved, we first measured food ingestion by 15 day-old flies using the capillary feeder (CAFE) method [38]. As shown in Figure 3A, the amount of food intake was similar between control and *nprl2*-mutant flies. Next, we counted the number of feces to determine the differences in the rate of food digestion between *nprl2*-mutant and control flies. Interestingly, *nprl2*-mutant flies generated markedly less feces than control flies (Figure 3B). The reduction in defecation in *nprl2*-mutant flies may be due to slow GI motility. To test this hypothesis, we assessed food distribution in the GI tract by measuring the content of blue food dye in the crop and gut immediately after 30 min of feeding. As shown in Figure 3C, the crops of *nprl2*-mutant flies contained more blue dye than the crops of control flies. Consistent with the finding that the total amount of food intake was similar between *nprl2*-mutant and control flies, the guts of *nprl2*-mutant flies contained less food than the guts of control flies (Figure 3D). Thus, the movement of food was much slower from the crop to the gut in *nprl2*-mutant flies. Similar to the mammalian stomach, the crop is a structure consisting of a complex array of valves and sphincters that contracts dynamically to move the food into the midgut. We also found that the crop contraction rate was significantly reduced in *nprl2*-mutant flies (Figure 3E), which suggested that the muscle, neuron, or other factors that control crop contraction were affected by *nprl2* mutation. These results suggested that GI tract dysmotility is related to slow food digestion in *nprl2*-mutant flies.

### The phenotypes of *nprl2*-mutant flies are related to TORC1 hyperactivation

**Figure 4:**
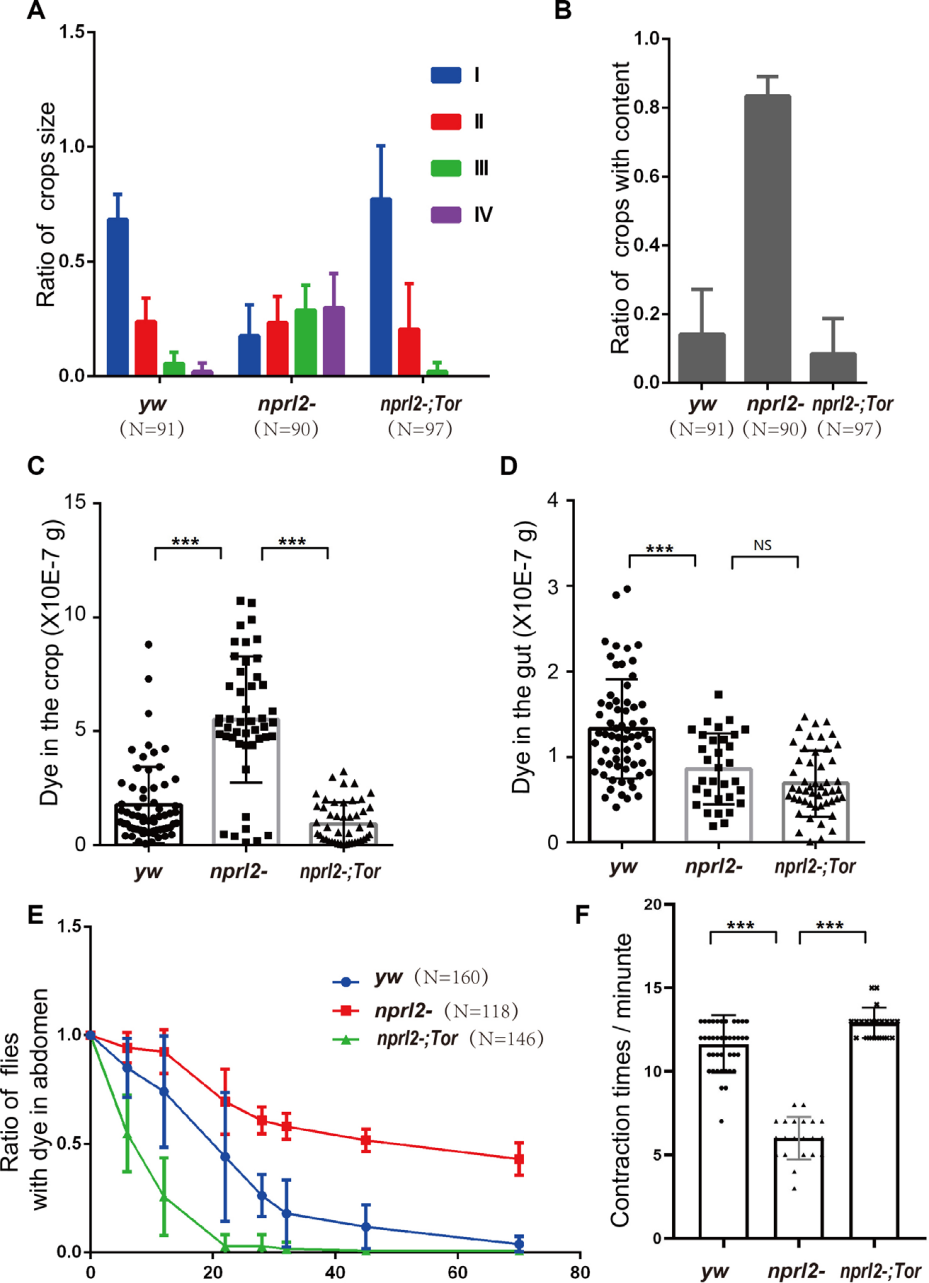
**Mutant one copy of *Tor* rescued the defects in digestive function in *nprl2* mutants.** Fifteen-day-old *yw*, *nprl2^1^*, and *nprl2^1^; Tor^A594V^/+* males were used for the following analyses. (**A** and **B**) The ratios of crop size and crops with food content were quantified as described in Figure 2. Error bars represent the SD from three independent experiments. (**C** and **D**) Flies were cultured in dyed food for 3 days and the dyed food concentration in the crop and gut was determined using a spectrophotometer. Error bars represent the SD of the indicated number of data points. (**E**) Flies were cultured on blue dyed food for 3 days and then transferred to standard food. The ratio of flies with blue dye in the digestive tube was calculated. Error bars represent the SD from four independent experiments. (**F**) Contractions of the crop were measured in dissected flies. Error bars represent the SD of the indicated number of data points. NS, not significant. ***P < 0.001. N is the total number of flies used.

As a component of the GATOR1 complex, Nprl2 inhibits TORC1 activity. We reasoned that the crop-emptying and GI tract motility defects in *nprl2*-mutant flies were related to hyperactivated TORC1. Thus, we placed one copy of the Tor mutation, *Tor^A948V^*, into *nprl2* mutants, which might decrease TORC1 activity in *nprl2*-mutant flies. We determined the TORC1 activity through detecting the phosphorylation of the downstream effector 4E-BP. The phosphorylated 4E-BP level was significantly increased in *nprl2* mutant, which could be rescued by mutant one copy of *Tor* to *Tor^A948V^* (Supplementary Figure 3). Interestingly, the size and food content of the *nprl2*-mutant crops were rescued by decreasing TORC1 activity (Figure 4A and 4B). We further aimed to determine whether food distribution in *nprl2* mutants was also rescued, by detecting the amount of dyed food in the crop and gut after 3 days of feeding. Consistent with the decreased crop size, the amount of dyed food in *nprl2*-mutant crops was also reduced in *Tor^A948V^* heterozygous flies (Figure 4C). Although the decrease in TORC1 activity in *nprl2* mutants did not rescue the smaller amount of food in the gut, the ratio of food distribution between the gut and the crop was comparable to control flies, which suggested that the abnormal food distribution was rescued (Figure 4C and 4D). Next, we determined the rate of food digestion by measuring the amount of dyed food expelled from the body. As shown in Figure 4E, one copy of mutant *Tor* greatly accelerated the expelling of food from the body in *nprl2* mutants. Interestingly, food was expelled at a greater rate from *nprl2;Tor^A948V^/+* flies than from control flies, which may be related to reduced food storage in the crop. In addition, the contraction rate in *nprl2* mutants with one copy of *Tor^A948V^* was also increased compared to control flies (Figure 4F). To further confirm the relationship between TORC1 activity and crop defects in *nprl2-* mutant flies, we treated flies with the TORC1-specific inhibitor, rapamycin. The food distribution defects were also rescued by rapamycin treatment (Supplementary Figure 4). Thus, our results suggested that hyper-activated TORC1 is related to the GI motility and food passage defects in *nprl2*-mutant flies.

### The *nprl2*-mutant flies display gut senescence

**Figure 5:**
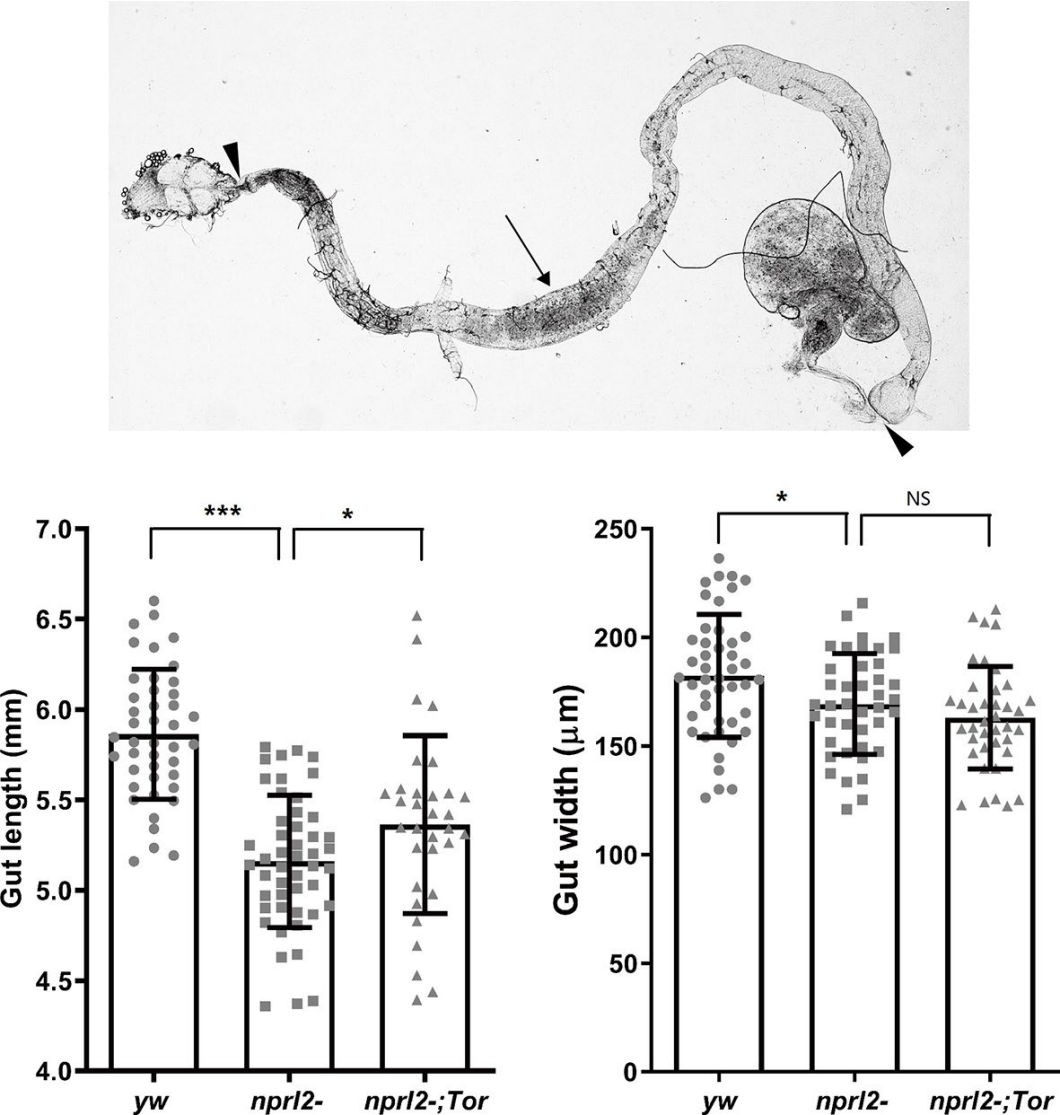
***nprl2* mutation decreased gut length and width.** Newly hatched male *yw*, *nprl2^1^*, and *nprl2^1^; Tor^A594V^/+* flies were cultured on standard food for 15 days and then dissected. Gut length (between the arrowhead) and gut width (arrow) were then measured. Error bars represent the SD of the indicated number of data points. *P < 0.05, ***P < 0.001. NS, not significant.

**Figure 6:**
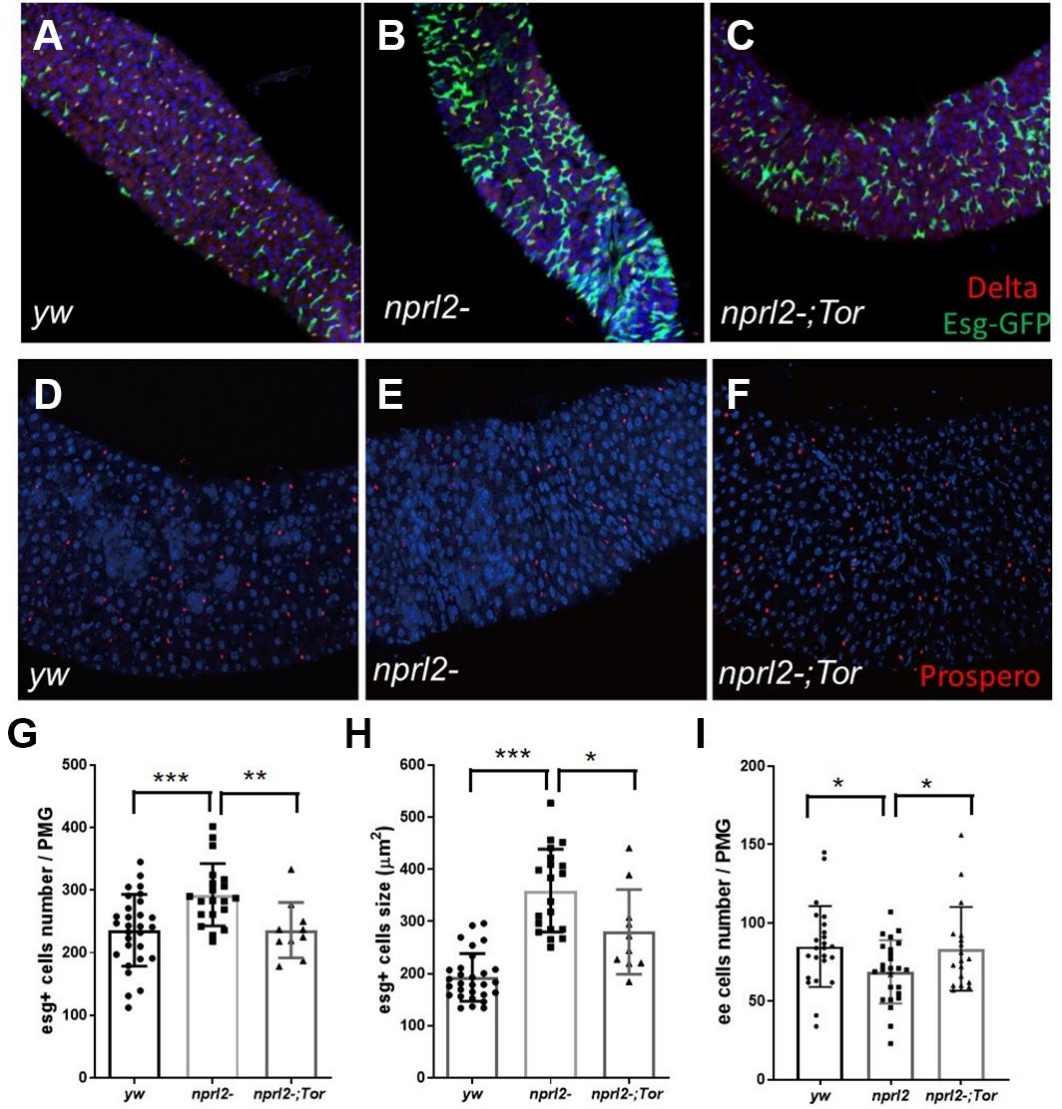
***nprl2* mutation affected ISCs and EE cells in the midgut.** (**A**–**C**) Newly hatched (**A**) *yw; esg-Gal4, UAS-GFP, tub-Gal80^ts^,* (**B**) *nprl2^1^; esg-Gal4, UAS-GFP, tub-Gal80^ts^* and (**C**) *nprl2^1^; esg-Gal4, UAS-GFP, tub-Gal80^ts^* / *Tor^A594V^* flies were cultured on standard food for 15 days at 18°C, then transferred to 29°C and cultured for a further 3 days. Flies were dissected and stained with anti-Delta antibody. (**D**–**F**) Fifteen-day-old (**D**) *yw,* (**E**) *nprl2^1^* and (**F**) *nprl2^1^; Tor^A594V^/+* flies were dissected and stained with an anti-Prospero antibody. (**G**) The number of Esg+ cells in the posterior midgut (PMG) was counted. (**H**) The size of Esg+ cells in the PMG was measured. (**I**) The number of Prospero+ cells in the PMG was counted. Error bars represent the SD of the indicated number of data points. *P < 0.05, **P < 0.01, ***P < 0.001.

To efficiently acquire nutrients, food movement and digestion in the GI tract must be regulated and coordinated properly. Therefore, we further performed phenotypical analysis of the midgut, which is comparable to the mammalian intestine. Midgut epithelium homeostasis, maintained by ISCs, is essential for the preservation of intestinal integrity. During aging and stress, ISCs are induced to proliferate and generate daughter cells, which will further differentiate into ECs and EE cells to replace the damaged or lost cells and remodel the gut. Thus, gut length is dynamic and is influenced by aging or stress [39, 40]. Here we found that the guts of *nprl2* mutants were markedly shorter and thinner than the guts of control flies at 15 d, which suggested that the *nprl2* mutants failed to generate enough epithelial cells and that epithelial homeostasis was impaired (Figure 5). Furthermore, overexpressed HA-tagged Nprl2 in the *nprl2*-mutant background increased the gut length and width in 15-day-old *nprl2* mutants (Supplementary Figure 5). Increased ISC proliferation and decreased terminal differentiation of ISC progeny is a hallmark of intestinal aging, which features the accumulation of polyploid cells expressing the ISC and enteroblasts (EB) marker, Esg. Previous studies have shown that loss of the tuberous sclerosis complex (TSC), another TORC1 inhibitor, results in ISC differentiation defects, with larger Esg+ cells and fewer EE cells, marked by Prospero expression [41, 42]. To test whether *nprl2* mutants also have accelerated gut aging and decreased stem cell maintenance, we determined the cell composition at the posterior midgut. The *nprl2*-mutant flies had a greater number of ISCs marked by Esg expression and fewer EE cells marked by Prospero expression than control flies, which indicated the dysfunction of ISC maintenance and differentiation in *nprl2* mutants (Figure 6). Interestingly, the gut length and cell composition phenotypes of *nprl2* mutants were both rescued by the decrease in TORC1 activity through mutant one copy of *Tor* to *Tor^A948V^* (Figures 5 and 6). These results suggested that the loss of epithelium maintenance is accelerated in *nprl2*-mutant flies through increased TORC1 activity.

Previous works have shown that the excessive proliferation and mis-differentiated ISCs are widely observed in the aged or damaged gut with a dysplastic phenotype [43, 44]. To monitor the proliferation of the ISCs, we used EdU method to detect the ratio of ISCs in S phase. Compared to only few ISCs labeled with EdU in control flies, large amount of ISCs in *nprl2* mutant were labeled (Supplementary Figure 6). This result suggests that *nprl2* mutant flies have more ISCs in proliferation state, which consistent with the hypothesis of *nprl2* mutant promotes gut aging.

**Figure 7:**
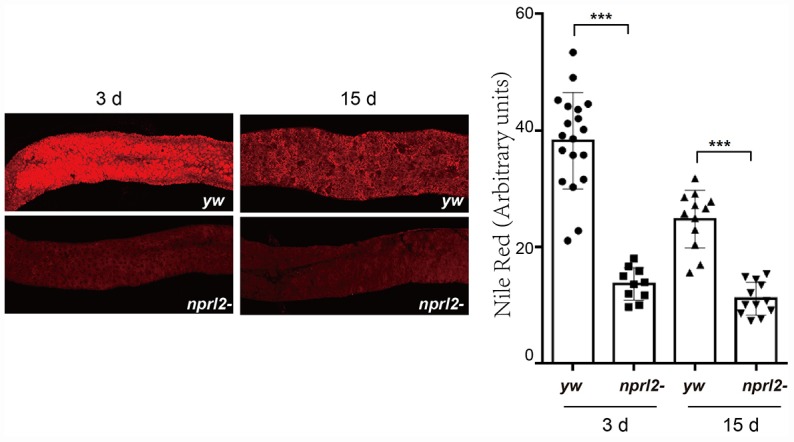
***nprl2* mutation decreased lipid storage in the midgut.** Newly hatched *yw* and *nprl2^1^* flies were cultured on standard food for 3 or 15 days and then dissected. The midgut was stained with Nile red. Error bars represent the SD of the indicated number of data points. ***P < 0.001.

Next, we evaluated other common features of the aging gut, including metabolic disorders. A reduction in lipid storage in aged fly guts has been demonstrated through lipid-associated dye staining [45]. We used Nile red to stain fly guts and found that the *nprl2*-mutant flies had much less lipid storage than control flies in the anterior midgut at both 3 and l5 days (Figure 7). Furthermore, the amount of lipids was less at 15 days than at 3 days in control flies, which was consistent with previous reports that lipid storage decreases with age. These results suggested that *nprl2* mutant promotes age-related metabolic disorder.

### The *nprl2*-mutant flies are sensitive to sodium dodecyl sulfate treatment

**Figure 8:**
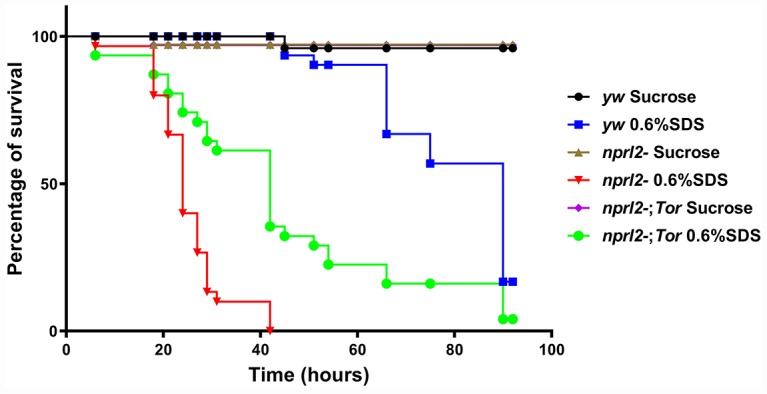
***nprl2* mutants were sensitive to SDS treatment.** *yw,* *nprl2^1^* and *nprl2^1^; Tor^A594V^/+* flies were fed with 5% sucrose or with 0.6% SDS. Flies were counted at the indicated time points. Survival curves of the flies are shown. Pairwise comparisons by the Mantel-Cox log rank test showed that survival curves of the three genotypes of flies fed 0.6% SDS were different from each other: *P* < 0.001 (*yw* vs *nprl2^1^*), *P* < 0.001 (*nprl2^1^* vs *nprl2^1^; Tor^A594V^/+*).

To assess the biological outcomes of the absence of Nprl2, we fed *nprl2*-mutant flies with the tissue-damaging agent, sodium dodecyl sulfate (SDS). SDS causes gut damage to flies and can be lethal [46]. When a low dose of 0.6% SDS in sucrose solution was given, the median lifespan of *nprl2^1^* flies (24 hours) was much shorter than the median lifespan of wild-type flies (90 hours, Figure 8). Increased vulnerability to a tissue-damaging reagent is consistent with the long staying of the food in the body. Interestingly, decreasing TORC1 activity through mutant one copy of *Tor* to *Tor^A948V^* could partially rescued the effects of SDS treatment in *nprl2* mutants, resulting in a median lifespan of 42 hours. Thus, these results suggested that Nprl2 protected the digestive tract from damage.

## DISCUSSION

Our study demonstrated the occurrence of abnormal crop size and digestive dysfunction with age. This age-related digestive disorder was reminiscent of GI motility and gastric emptying defects in the elderly, and thus underlines the relationship of aging and GI dysmotility. Mutation of *nprl2* contributed to age-related crop distension and digestive dysfunction, and this could be rescued by decreasing TORC1 activity. The association between TORC1 activity and many age-related diseases remains elusive, but the work presented herein provides a possible mechanism for the promotion of digestive organ senescence by TORC1.

### Aging affect crop size and digestive function in *Drosophila*

Base on the functional and structural similarity between *Drosophila* digestive tract and mammalian GI tract, the *Drosophila* intestine is widely used to study the mechanism of ISC proliferation and differentiation, barrier function, immune response and metabolic change during aging and stress [24, 30, 47]. Compared with the great progress had made on the midgut, little is known about the function of the *Drosophila* crop, which is analogous to the mammalian stomach. Some clinical studies have shown that a decreased rate of gastric emptying may be relevant to many symptoms that occur in the elderly [19, 20, 23]. Here, we found that the occurrence of a swollen crop and food accumulation in the crop were also correlated with age in *Drosophila*. Consistent with the gastrointestinal dysmotility seen in the elderly, crop contraction rate was also decreased in *nprl2*-mutant flies. These results suggested that aging may affect digestive function in the *Drosophila* crop, in a similar manner to mammals and this provide a good model for age-related GI dysmotility research. However, it should be noted that the swollen crop may have been due to defects in crop contraction and/or midgut digestion, which affect the passage of food.

### Hyperactivated TORC1 accelerates aging and age-related digestive disorders

Recently, two mutants, *drop-dead* (*Drd*) and *Beadex*, were shown to have enlarged crops and reduced fecal deposition at a very early stage and the crop is almost empty. This suggests that the *Drd* and *Beadex* genes are essential for the development of specific tissue required to maintain crop function [36, 48]. Moreover, *Drosophila* C virus infection can also induce a distended crop, possibly by affecting crop muscle function [49]. Here, we found that mutation of *nprl2*, which encodes an inhibitor of TORC1, also resulted in an enlarged crop. Interestingly, the distended crops in *nprl2* mutants were accompanied by food accumulation in the crops and this phenotype emerged a few weeks after eclosion, which suggested that the crop defect was due to premature aging, rather than improper development. By using a blue dye, we further showed that mutation of *nprl2* affected the rate of food movement and food distribution in the digestive tract. Furthermore, decreased TORC1 activity through genetic manipulation, mutant one copy of *Tor* to *Tor^A948V^*, or pharmacy treatment, using TORC1 specific inhibitor rapamycin, rescued the crop phenotypes of distend size, food accumulation and contraction rate in *nprl2* mutant, which suggests that hyper TORC1 activity promotes age-related GI dysfunction.

Although the phenomenon that decreasing TORC1 activity increase lifespan in many organisms, the mechanism remains still unclear. One important question is whether activated TORC1 can promote aging. Nprl2 mediates amino acid levels and acts as an important TORC1 inhibitor. Our results showed that *nprl2*-mutant flies had shorter lifespans and age-related GI dysfunction. In addition, our previous works showed that *nprl2*-mutant flies display some defects such as female fecundity, energy storage and locomotion [13, 34, 50]. These characters might result from defects in other tissues and might also contribute to the decrease of lifespan. Therefore, *nprl2*-mutant flies may serve as an important tool to address questions regarding the mechanism of TORC1 activity and aging.

### Hyperactivated TORC1 promotes midgut aging

TORC1 activity plays important roles in ISC maintenance. Mutation of the TORC1 inhibitor, TSC2 results in defects in ISC division and differentiation, which impairs tissue homeostasis and increases the susceptible of flies to tissue damage [41]. On the contrary, inhibiting TORC1 activity through rapamycin treatment delays midgut aging [51]. Here, we found that the *nprl2* mutant displayed an increase in ISC proliferation and a decrease in the number of differentiated EE cells, which was consistent with a previous report on mutations in the TORC1 inhibitor, TSC1 [42]. Furthermore, we found that lipid storage decreased in *nprl2*-mutant midguts, which can also be seen in aged flies [45]. Recently, it was reported that rapamycin treatment modulates tissue aging and protein aggregates, which suggests that the effects of TORC1 activity on aging may be mediated by autophagy [52]. Notably, autophagy is inhibited in GATOR1 mutants [34], which may be related to the aging and age-related digestive dysfunction seen in *nprl2*-mutant flies. Recently, a theory, proposed by Mikhail Blagosklonny, suggests that the hyperfunction of TORC1 and other nutrient-sensitive signaling is the cause of aging [53, 54], which was tested in *C. elegans,* a model system that also develop age-related pathology including digestive dysfunction and tumor formation [55, 56]. Here the age-related digestive dysfunction in *nprl2* mutant flies might be contributed by the hyperfunction of TORC1.

In conclusion, hyperactivated TORC1 in *nprl2*-mutant flies decreased lifespan and promoted the occurrence of age-related crop enlargement, digestive dysfunction, and midgut senescence. Our findings suggested that Nprl2 is essential for the maintenance of GI function during aging, by suppressing TORC1 activity.

## MATERIALS AND METHODS

### Fly stocks

The stocks *yw*, *nprl2^1^*, and *UASp-3×FLAG-3×HA-Nprl2*, have been described previously [34]. *y, w; Tor^A948V^/CyO* was kindly provided by Thomas P. Neufeld [57]. *y w; esg-Gal4, UAS-GFP, tub-Gal80^ts^* (TB00137) were obtained from Tsinghua Fly Center (Beijing, China). Flies were cultured at 60% humidity in a temperature-controlled incubator at 25 °C, with a 12 h on/off light cycle, unless otherwise noted. Only male flies were used in this study. The standard food consisted of 1% agar, 3% brewer’s yeast, 1.9% sucrose, 3.8% dextrose, and 9.1% cornmeal (all concentrations in wt/vol). The dyed medium was prepared using standard medium with FD&C Blue Dye No. 1 (SangoTech, Shanghai, China) added at a concentration of 0.5% wt/vol. For rapamycin treatment, rapamycin (LC Laboratories, Woburn, MA, US) was dissolved in methanol and mixed into the media when preparing food vials, to a final concentration at 200 μM. For SDS treatment, SDS was dissolved in 5% sucrose to a final concentration at 0.6%. For ISC experiments using *esg-Gal4, UAS-GFP, tub-Gal80^ts^*, fly crosses were performed at 18°C and newly enclosed flies of the appropriate genotypes were kept at 18°C and then transferred to 29°C for 3 days before dissection.

### Western blots

Male *Drosophila* flies were homogenized in RIPA buffer containing complete protease inhibitors and phosphatase inhibitors (Roche, Basel, Switzerland). Western blotting was performed as described previously [13]. Antibodies were used at the following concentrations: Rabbit anti-p4E-BP antibody (Cell Signaling Technology, Danvers, MA, USA) was used at 1:500 and mouse anti-α-tubulin antibody was used at 1:10000 (Jackson Immuno-Research, West Grove, PA, USA). Band intensities were quantified using Image J software (NIH, Bethesda, MD, USA).

### Crop size and gut length quantification

Enclosed male flies were collected and cultured on standard medium until the day of analysis. The *Drosophila* gut was dissected in phosphate-buffered saline (PBS) media, fixed for 20 min in 4% formaldehyde, and then washed twice and sealed with nail oil. Images were captured using a compound microscope. Gut length and crop area were quantified using Image J software.

### Food retention assay

Flies were allowed to consume dyed food for 72 hours prior to quantification. After 72 hours, the dye was clearly observed in the digestive tract. The flies with dye in the gut were fed standard food and the ratio of flies with observed dye in the digestive tract was determined at the indicated time points.

### Dyed food distribution assay

For immediate food distribution, flies were starved in complete starvation medium (PBS with 1% agarose) for 10 hours and then fed dyed food for 30 min prior to quantification. To determine stable-state food distribution, flies were fed dyed food for 72 hours prior to quantification. The fly gut was dissected, and the crop was carefully separated from the entire gut. Collected tissues were homogenized in 100 μL of PBS. The absorbance values at 630 nm were measured using a spectrophotometer. The dye concentration in crops and guts was calculated by comparison with a standard curve.

### Crop motility assays

The crop motility assay was performed as previously described [48]. The male fly was pinned in a dissecting dish under PBS and the ventral cuticle was removed in order to view the crop. The contractions of a crop lobe were recorded from each fly for five repeats with each one is 30 second (with 30 second intervals between each recording period) and then the five counts were averaged.

### Capillary Feeder (CAFE) assay

The CAFE assay was modified from previously described [38]. Briefly, 10 male flies were put in the inner chamber to get the liquid food (0.5% Blue dye in 5% sucrose media) for 24 hours and the amount of food loss was calculated. Each experiment included an identical CAFE chamber without flies to determine evaporative losses, which were subtracted from experimental readings.

### Defecation rate

The defecation rate detection was modified from previously described [37]. Flies were allowed to consume dyed food for 3 days prior to quantification. The male flies were then transferred to empty cleared vials (10 flies per vial) for 1 hour and the number of blue fecal spots on the vial counted. The rate of defecation was calculated as the number of spots per fly per hour.

### Nile red assay

*Drosophila* guts were dissected in PBS, fixed in 4% formaldehyde for 20 min, and then washed three times with PBS. The guts were incubated with Nile red for 30 min, washed twice, and mounted with ProLong ® Gold Antifade Reagent containing DAPI (Invitrogen, Carlsbad, CA, USA).

### EdU labeling assay

The EdU labeling assay was modified from previously described with a Click-iT EdU Alexa Fluor 594 Imaging Kit (Thermo Fisher Scientific, C10339) [58]. Male flies were fed with 100 μM EdU and 5% sucrose for 24 hr. The EdU incorporation in gut was visualized by Click reaction according to the manufacturer’s instruction.

### Immunofluorescence confocal microscopy

Immunofluorescence staining was performed as previously described [50]. The mouse anti-Prospero antibody (Developmental Studies Hybridoma Bank, University of Iowa) was used at 1:100. The mouse anti-discs large antibody (Developmental Studies Hybridoma Bank, University of Iowa) was used at 1:100. Alexa Fluor-conjugated anti-mouse secondary antibodies (Invitrogen) were used at a dilution of 1:1,000. Nuclei were visualized by staining with DAPI (Invitrogen). Images were acquired using a Zeiss 880 confocal microscope (Zeiss, Oberkochen, Germany) and fluorescence was quantified using Image J software.

## Supplementary Material

Supplementary Figures
