# Supplementary material for: The TORC1 inhibitor Nprl2 protects age-related digestive function in Drosophila
Source: Aging (Albany NY). 2019 Nov 11;11(21):9811–28. doi: 10.18632/aging.102428 (PMC6874466; doi:10.18632/aging.102428)
Supplement: Supplementary Figures [file aging-11-102428-s001.pdf]

SUPPLEMENTARY FIGURES

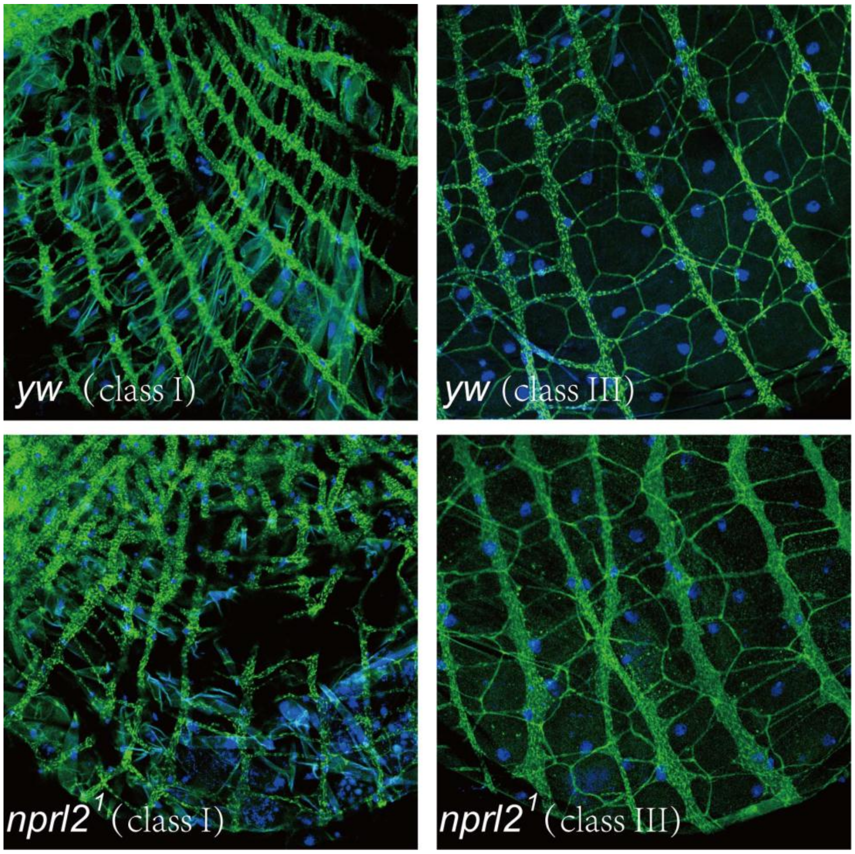

Supplementary Figure 1. The enlarged crops were caused by stretching. The small (class I) and enlarged (class III) crops from 15-day-old *yw* and *nprl2*[1] flies were stained with an anti-Discs large (green) antibody and DAPI (blue).

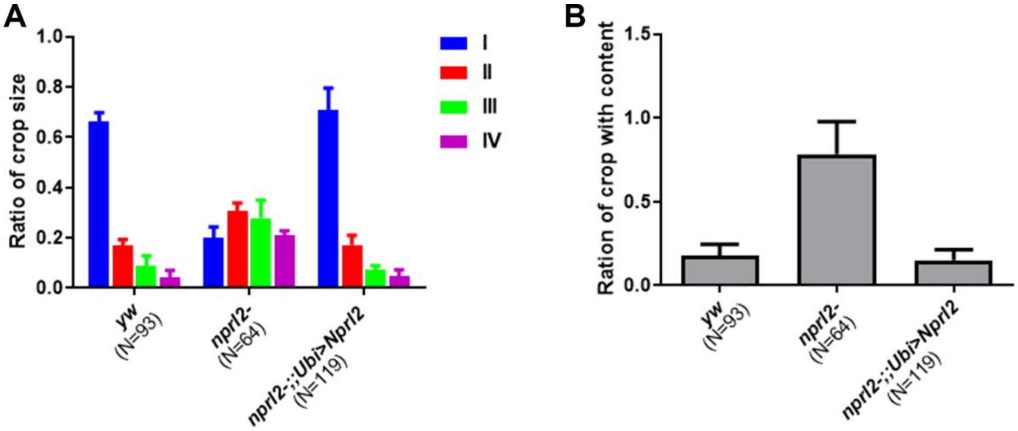

Supplementary Figure 2. Overexpressing Nprl2 rescued the digestive dysfunction of the *nprl2* mutant. Newly hatched *yw, nprl2*[1] and *nprl2*[1]*; Ubi-GAL4/UAS-HA-FLAG-Nprl2* flies were cultured in standard food for 15 days. The ratios of crop size (**A**) and crops with food content (**B**) were quantified as described in Figure 2. Error bars represent the SD from four independent experiments. N is the total number of flies used.

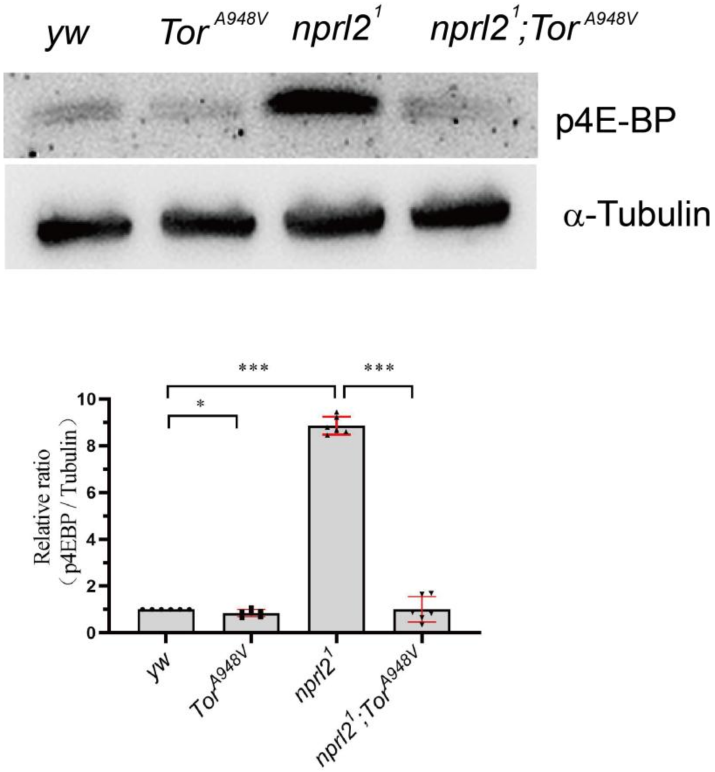

Supplementary Figure 3. Mutant one copy of *Tor* to *Tor*^{A594V} decreased TORC1 activity. (**A**) Protein lysates from 15-day-old *yw*, *Tor*^{A594V}*/+, nprl2*^{1} and *nprl2*^{1}*; Tor*^{A594V}*/+* flies were used for western blotting to determine the levels of the downstream TORC1 effector, phospho-4E-BP. (**B**) The relative phosphorylated 4E-BP levels were shown. Error bars represent the SD of the indicated number of data points. **P* < 0.05, ****P* < 0.001.

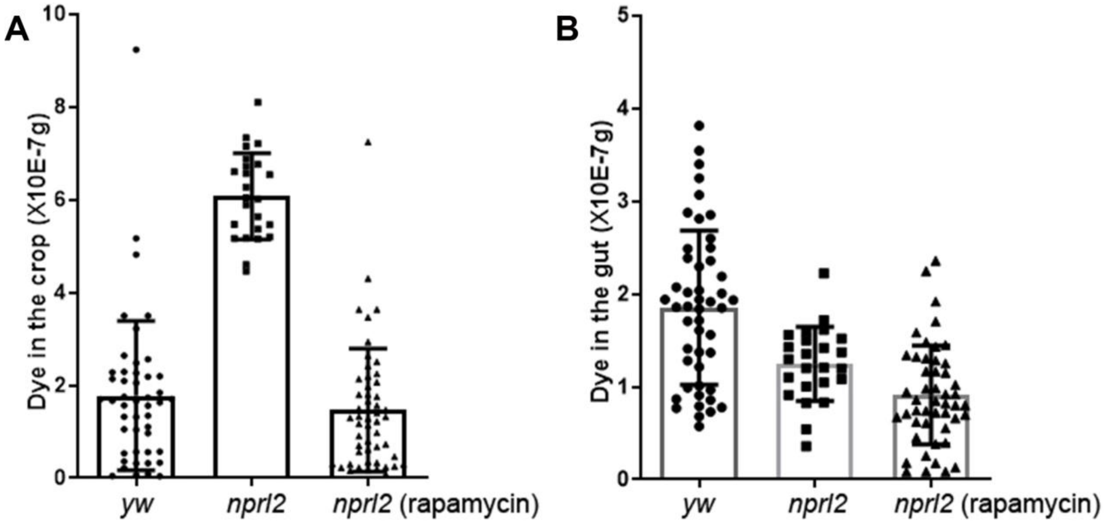

Supplementary Figure 4. Rapamycin treatment rescued the food distribution defects of the *nprl2* mutant. *yw* and *nprl2*^{1} flies were cultured in standard food or standard food with 200 μM rapamycin for 15 days and then transferred to dyed food for a further 3 days. The concentration of dyed food in the crop (**A**) and gut (**B**) was determined using a spectrophotometer. Error bars represent the SD of the indicated number of data points.

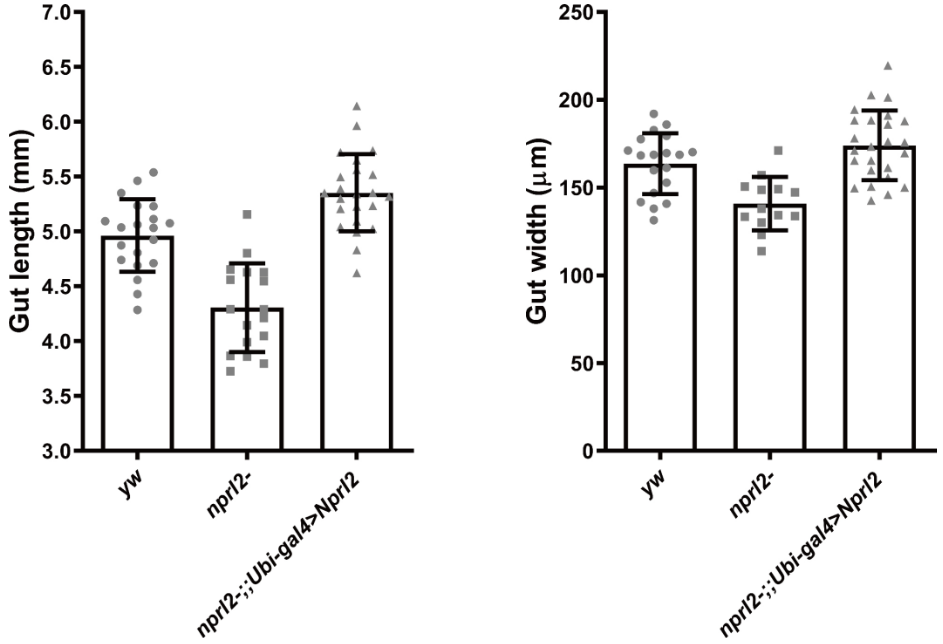

Supplementary Figure 5. Overexpressing Nprl2 rescued the gut length and width phenotypes of the *nprl2* mutant. Newly hatched *yw, nprl2*[1], and *nprl2*[1]*; Ubi-GAL4/UAS-HA-FLAG-Nprl2* flies were cultured in standard food for 15 days. Gut length and width were then measured. Error bars represent the SD of the indicated number of data points.

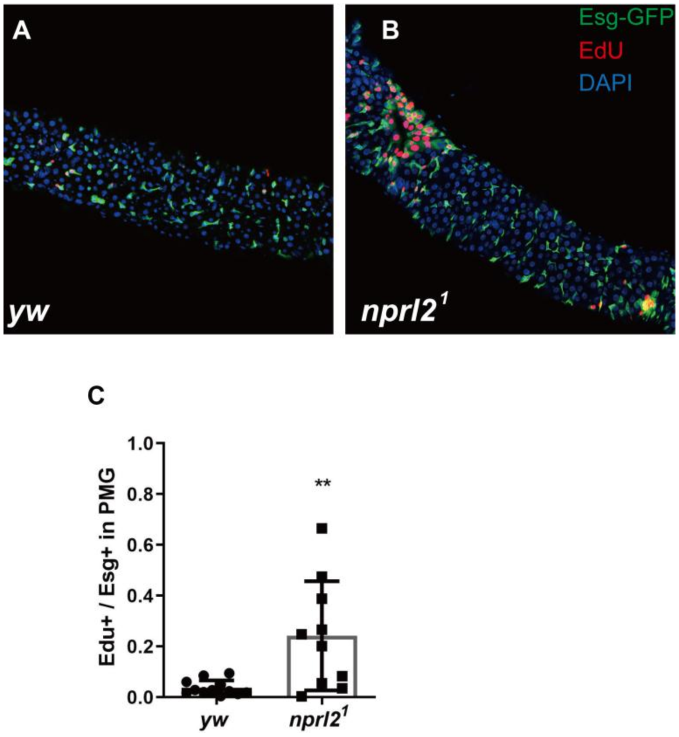

Supplementary Figure 6. The ISCs proliferation increased in *nprl2* mutant flies. Newly hatched (**A**) *yw; esg-Gal4, UAS-GFP, tub-Gal80*[ts] and (**B**) *nprl2*[1]*; esg-Gal4, UAS-GFP, tub-Gal80*[ts] flies were cultured on standard food for 15 days at 18°C, then transferred to 29°C and cultured for a further 3 days. Flies were treated with EdU labeling following Materials and Methods description. (**C**) The ratio of Esg+ cells with EdU labeling were counted. Error bars represent the SD of the indicated number of data points. $^{**}P < 0.01$.
